# Blood Pressure in Surgery

**Published:** 1904-04

**Authors:** 


### BOOK REVIEWS.

Blood Pressure in Surgery. An Experimental and Clinical Research. The Cartwright Prize Essay for 1903. By George W. Crile, A. M., M. D., Professor of Clinical Surgery, Western Reserve Medical College, Cleveland. Octavo, pages 422. Illustrated. Philadelphia and London: J. B. Lippincott Co. 1903.

The wide diversity of opinion upon the causes of low blood-pressure in surgical cases and of the methods of combatting the same, led the author to institute an extensive series of investigations upon the subject, with the view to determine among other things the action of the more common agents used for stimulation; to determine what produces lowered blood-pressure, whether exhaustion of the bloodvessels, of the heart, of the vasomotor, of the cardiac center, or of the respiratory center; whether it be an exhaustion or suspension of function or the passages of plasma through the vessel walls, and the like. Among the drugs employed were included, alcohol, nitroglycerin, amylnitrite, digitalis, strychnin, saline infusion, adrenalin, and morphine; and the conclusions arrived at after most painstaking and exhaustive experimentation are both surprising and instructive.

Briefly summed up they are as follows: alcohol was found invariably to be a depressant, and the more pronounced the shock the more depressant its action. Nitroglycerin and amylnitrite also almost uniformly depressed the blood-pressure and increased the shock. Digitalis increased the blood-pressure in normal doses, but it seemed on the average that the cases of shock so treated did not live as long as the controls. Strychnin in sufficient amount caused rise in blood-pressure, the rise being proportional to the shock, but in every degree of shock after administration of therapeutic doses the animals finally passed into deeper shock.

Whenever the foregoing drugs were given in conjunction it was found that digitalis and strychnia supplemented each other very well and a steady and well sustained rise in pressure followed their administration. When nitroglycerin was used, whether after or simultaneously with digitalis and strychnin, there was invariably a fall, in some instances compensation being partial, in others, complete. Alcohol was always found to be a progressive depressant.

The author, summing up his conclusions upon these points, says, "one might be able to so adjust the dosage of strychnin, digitalis, amylnitrite and nitroglycerin that they might neutralise each other in their immediate effect upon the blood-pressure. The experimental data show that the treatment of shock by the most commonly employed stimulants—namely, strychnin, brandy, digitalis and nitroglycerin, produced systemic confusion in the animal. Saline infusion always caused a rise in pressure, which was usually gradual and sustained in proportion to the amount of shock present when it did not exceed a moderate degree; in the more pronounced cases the rise was not so marked or well sustained. The blood count and hemoglobin estimations showed that the blood was not much diluted by the saline. Adrenalin, in the normal animal, in every degree of shock and collapse, and under all conditions, caused a rise in blood pressure following its exhibition. Morphine given before etherisation gave better results than etherisation alone, and the two formed a combination under which more extensive operations and procedures over a longer period of time were possible, than by means of ether or chloroform anesthesia alone."

Many interesting and instructive observations upon cocainisation of the stellate ganglia to prevent shock, upon the effect of pressure upon the circulation, including the use of the pneumatic rubber suit upon patients to maintain a constant pressure in prolonged and extensive operations; many clinical observations upon blood-pressure in which the various methods of estimation are clearly and exhaustively given, and finally the conditions of blood-pressure existing under various normal conditions of the body and in varied pathological states, and a study of the changes in the blood-pressure during surgical operations conclude the pages of this most admirable work.

One thing deserves especial mention, and that is, the differentiation which the author makes between shock and collapse; inasmuch as the therapeutic indications are so different in the two conditions. Crile believes surgical shock to be an exhaustion of the vasomotor centers, in which the heart-muscle, the cardiac centers, and the respiratory centers are only secondarily involved; whereas collapse is due to the suspension of the cardiac or of the vasomotor mechanism, or to hemorrhage; and the desirability of accurate differentiation is emphasised by his conclusions upon the treatment of the two conditions. For example, in shock, therapeutic doses of strychnin are inert, physiologic doses are dangerous or fatal; if not fatal increased exhaustion follows. Stimulants of the vasomotor center are contraindicated in shock; cardiac stimulants have but a limited range of possible usefulness, and may be injurious. In collapse, stimulants may be useful, because the centers are not exhausted. Saline infusion in shock has a limited range of usefulness; in collapse it may be effective; the blood tolerates but limited dilution with saline solution; elimination takes place through the channels of absorption; its accummulation in the splanchnic area may be sufficient to fix the diaphragm and the movable ribs, causing death by respiratory failure. In shock, it raises, but cannot sustain the blood-pressure.

Adrenalin acts upon the heart and bloodvessels, raising the blood-pressure in the normal animal; is rapidly oxidised by the solid tissue and by the blood; its effects are fleeting; it should be given continuously; its clinical value still remains unproved. The pneumatic rubber suit provides an artificial peripheral resistance, without injurious side effects, and gives a control over the blood-pressure within a range of 25-60 mm. mercury. Crile says that "by the combined use of artificial respiration rhythmic pressure upon the thorax over the heart and the infusion of adrenalin, animals which were apparently dead as long as 15 minutes were resuscitated; by the same method, with the addition of the rubber suit, a patient who from fatal injury of his brain had been conventionally dead for 9 minutes was partially resuscitated for 32 minutes, during which time a strong heart-beat was noted and he was able to move his head."

The work, as a whole, is a monument to the industry and intelligence of its author, and should be read and digested by every practising physician and surgeon. J. P.

